# Effect of salinity level on TSH and thyroid hormones of grass carp, *Ctenophayngodon idella*

**Published:** 2013

**Authors:** Rahim Peyghan, Ala Enayati, Mostafa Sabzevarizadeh

**Affiliations:** *Department of Clinical Sciences, Faculty of Veterinary Medicine, Shahid Chamran University of Ahvaz, Ahvaz, Iran.*

**Keywords:** Grass carp, Salinity, Serum, Thyroid hormones, Thyrotropin

## Abstract

Thyroid hormones (T_3_, T_4_) have marked effect on body metabolism and in controlling osmoregulation activity in fish. The aim of this study was to determine the effect of water salinity changes on thyroid hormones level and thyroid-stimulating hormone (TSH) of grass carp. For this purpose 120 grass carp were divided randomly in to four groups (10 fish in each group and three replicates per treatment). Three groups were held in three different salinities at concentrations of 4, 8 and 12 g L^-1^. The fourth group was reared in fresh water and considered as control. After three weeks blood samples were collected from the caudal peduncle vein. Then serum was separated and serum thyroid hormones and TSH were measured by LISA on Microwell plates. Our results indicated that the average of T_3_ levels in 4, 8 and 12 g L^-1^ groups were 0.43 ± 0.11, 0.22 ± 0.04 and 0.21 ± 0.04 μg dL^-1^, respectively. T_3_ levels in all experimental groups were significantly lower than those of control group (*p* < 0.05). Serum T_4_ level in 4, 8 and 12 g L^-1^ groups were 0.29 ± 62955/40.06, 0.24 ± 43129/50.05 and 2.85 7958/± 05768/40.55 μg dL^-1^, respectively. Thyroxine level was significantly higher only in 12 g L^-1^ group in comparison with the control and other experimental groups (*p* < 0.05). Thyroxine level in other groups had not any significant difference with the control group (*p* > 0.05). The level of TSH in salinities of 4 and 8 g L^-1^ groups was significantly higher than that of control group (*p* < 0.05). The results showed that increasing water salinity can have significant effect on thyroid activity by decreasing T_3_ and increasing T_4_ level in serum of grass carp in experimental condition.

## Introduction

Salinity stress is one of the most common stresses in freshwater aquatic animals that usually occurs and if prolonged, can lead to reduction of production efficiency or fish death. Carps and other bonny fishes are able to regulate the ionic concentration of the internal body fluids within narrow ranges. In many studies, the exposure of freshwater fish to high salinities (salinity stress), is able to change the internal osmolarity of the plasma allowing an influx of water.^1^^-^^4^ Different hormones control the activities of the organs involved in osmoregulation, which maintain water and mineral balance in different salinity.^5^ Thus, conditions that affect or disrupt the ionic homeostasis, cause changes in hormone levels involved in osmoregulation as a response to control the ion transport capacity.^6^^-^^8^ The thyroid hormones, thyroxine (T_4_) and tri-iodothyronine (T_3_) are products of the thyroid gland in all vertebrates. Thyroid stimulating hormone (Thyrotropin, also known as TSH), T_4_ and T_3_ are the principal hormones secreted from the hypothalamic-pituitary-thyroid (HPT) axis; produce a wide range of physiologic actions in fish. Thyroid hormones of fish are small molecules very similar to other vertebrates. They are called T_4_ and T_3_ in fish. Tri-iodothyronine is the most effective biological thyroid hormone in fish.^9^ Thyroid hormones are important to control development, growth, metabolism and osmoregulation in fish and often do these activities in association with other hormones such as cortisol and growth hormone.^10^ Grass carp as a freshwater fish may be exposed to salinities higher than normal range. In some parts of Khuzestan province water salinity of fish ponds may be higher than usual in some months of the year. Since there are few studies on the thyroid hormones of grass carp, it was necessary to study the normal level as well as the effect of increase salinity on thyroid hormones level in grass carp.

## Materials and Methods

**Experimental groups. **In this study 120 grass carp (100 to 120 g weight range) were divided randomly into four groups (10 fish in each group and three replicates per treatment). Three groups were held in three different salinities at concentrations of 4, 8 and 12 g L^-1^. The forth group was reared in freshwater (1 g L^-1^) and considered as control. The gradual increase of salinity in experimental groups was done in 3-4 days. All other physicochemical parameters of water and feeding level were identical for all groups.

**Sampling and measurements. **After three weeks, the fish were anesthetized with 100 mg L^–1^ MS-222 (Sigma- Aldrich, *St. Louis*, MO, USA (by short term bath method and blood samples were collected from all fishes. For blood collecting, bleeding was done from the caudal peduncle vein. Blood samples were immediately transferred to sterile tubes and the serum was separated by centrifugation. In this study, T_3_, T_4_ and TSH were measured by enzyme linked immunosorbent assay (ELISA) method. Serum free T_4_ (fT_4_) level was measured manually by ELISA using commercially available kit (Delaware Biotech Inc., Heidelberg, Germany) according to the manufacturer’s instructions. For total T_3_ and TSH concentration in serum, immune enzymatic colorimetric method was used for quantitative determination using DiaMetra kit (DiaMetra Co., Milano, Italy) according to the manufacturer’s instructions. Absorbance was read at 450 nm in ELISA reader (Synergy HT, Winooski, VT, USA). 

**Statistical analysis. **Means of each parameter and changes were measured and compared in all groups by one way analysis of variance. The data were analyzed by SPSS (Version 16, SPSS Inc., Chicago, IL, USA) and the differences were considered significant at level of *p* < 0.05.

## Results

According to our results, the average of serum T_4_, T_3_ and TSH in control and 4, 8 and 12 g L^-1^ salinity groups were showed in Table1. The of level T_4_ was significantly higher only in 12 g L^-1^ group in comparison with control and the other experimental groups (*p* < 0.05). The level of T_4_ in other groups did not have any significant difference with control group (*p* > 0.05). The level of T_3_ in all experimental groups were significantly lower than the control group (*p *< 0.05). The level of TSH in 4 and 8 g L^-1^ salinity groups was significantly higher than control group (*p* < 0.05). 

**Table 1:** The average of serum thyroid hormones in grass carp in experimental groups and the control (Mean ± SE).

**پارامتر** **Group**	**T**_4 _ **(µg d**L^-1^**)**	**T**_3 _ **(µg d**L^-1^**)**	**TSH (µg d**L^-1^**)**
**گروه شاهد** **Control**	0.91 ±76328/3 0.19^a^	1.62 ±62068/2 0.23^a^	1136/0 ±0.26000.13 ± 0.01^a^ 03303/0
**4 (g L** ^-1^ **)** **گروه4گر م**	ᵅᵇ0.29 ±62955/4 0.06^a^	ᵇ9514/6 ±0.380.43 ± 0.10^b^	0.32 ± 0.05b 03169/0
**گروه 8گرم** **8 (g L** ^-1^ **)**	ᵇ0.24 ±43129/5 0.05^a^	ᵇ0.22 ± 0.04^b^ 90851/1	ᵅ0.30 ± 0.04^b^ 03102/0
**گروه12گرم** **12 (g L** ^-1^ **)**	2.85 7958/±05768/4 0.55^b^	ᵅ0.21 ± 0.04^b^ 44013/40.07	1242/00.25 ± 0.02^a^ 4333/ 0±

ab Different letter in each column show significant difference between each hormone concentration (*p* < 0.05).

## Discussion

Salinity changes can be stressful and may affect hemostasis of the fish. Numerous experiments on animals demonstrated that blood concentrations of thyroid hormones are increased during early exposure to stressors such as high ambient temperatures and excitement stress.^11^ Environmental factors such as day length, lunar cycle events, temperature, pH, salinity, and nutrition are all implicated in stimulating thyroid activity.^12^ In some fish species, thyroid hormones support the action of growth hormone and cortisol in promoting seawater acclimation.^13^ In our study we observed that with increasing salinity at concentrations of 4, 8 and 12 g L^-1^, serum T_3_ level in these three groups was decreased significantly in comparison with the control group. This decrease in T_3_ could be due to decreased hormone decomposition and delay or decrease of hormone secretion.^11^

 Serum T_4_ level in the group with 12 g L^-1^ salinity was increased significantly in comparison with other groups. The level of TSH in the groups with 4 and 8 grams per liter salinity were significantly higher than that of control group. Parker and Specker investigated the effect of salinity and temperature on whole-animal thyroid hormone levels in larval and juvenile striped bass, *Morone saxatilis.* Their results revealed that higher salinities increased T_4_ levels in fish larvae. They suggest that thyroid hormones may mediate the beneficial effects of salinity on larval striped bass growth and survival.^14^ Sampaio and Bianchini investigated salinity effects on osmoregulation and growth of the euryhaline flounder *Paralichthys orbignyanus,* and suggest that the lower growth rate exhibited by *P*. *orbignyanus* in fresh water could be due, at least partially, to a higher energy expenditure associated to a higher branchial Na^+^, K^+^ -ATPase activity in this environment.^3^

Most studies that involve exposing anadromous salmonid fishes to seawater report an increase in gill Na^+^ K^+ ^-ATPase activity suggesting that it is an integral part of their successful acclimation.^15^ Rejitha *et al*. investigated short-term salinity acclimation demands of thyroid hormone action in the climbing perch *Anabas testudineus *Bloch. Their results suggest that following transfer of fish to 20 ppt salinity for a day after transient salinity changes, plasma T_4_ was elevated, and plasma T_3_ decreased whereas plasma cortisol remained unchanged. The levels of these hormones, however, returned to basal levels when these fish were kept for a prolonged acclimation of three weeks. Their results indicate that salinity acclimation in climbing perch demands thyroid hormone secretion and its action and not cortisol as part of coordinating the acclimation processes in the early phase of salinity acclimation.^16^ In other experiment *Acanthopagrus latus* was held in different salinities and results indicated that thyroid hormone levels in plasma (T_3_) 12 hr after the salinity change compared to the control samples showed a small rise that quickly return to normal.^17^ Saunders *et al*. studied the effect of orally administered 3, 5, 3′-triiodo-L-thyronine on growth and salinity tolerance of Atlantic salmon (*Salmo salar*). They found that T_3_ treatment significantly increased salinity tolerance in mature males but not in immature fish.^18^ Klaren *et al*. investigated the effect of acclimation to low salinity water of gilthead seabream (*Sparus auratus*) on the activities of thyroid hormone-metabolizing enzymes in gills, kidney, and liver. The data reveal that following acclimation to low salinity water, the plasma free T_4_ concentration increases 2.5 fold, and outer ring de-iodination activities towards T_4_, 3,5,3′-tri-iodothyronine and 3,3′,5′-tri-iodothyronine (reverse T_3_ also known as rT_3_) in the gills are reduced by 20-32%.

Their results substantiate that thyroid hormones are involved in *S. auratus* osmoregulation, and that the gills are well equipped to play an important role in the modulation of plasma hormone titers.^19^ McCormick and Saunders showed that in juvenile Atlantic salmon (*Salmo salar*) following acute exposure to seawater (30 ppt), plasma T_4 _was increased 80% in the first 6 hr, declined to initial levels after 24 hr, and remained stable for 18 days, thereafter. In nonsmolts, plasma T_4_ did not rise immediately after exposure to seawater, fell slightly after 2 days, and remained low for 18 days. Plasma T_3_ of smolts and non-smolts was not affected by acute exposure to seawater.^20^ In other experiment *Solea senegalensis* was acclimated to seawater (38 ppt) and that were transferred and allowed to acclimate to different salinities (5, 15, 38 and 55 ppt) for 17 days. Plasma cortisol had increased, whereas plasma fT_4_ levels were decreased in animals that were transferred to salinities other than SW. The inverse relationship between plasma cortisol and fT_4_ suggests an interaction between these hormones during osmoregulatory period.^21^ Iwata *et al*. studied the effects of T_4_, growth hormone and cortisol on salinity preference of juvenile Coho salmon (*Oncorhynchus kisutch*). Fish treated with T_4_ or with ovine growth hormone showed a significantly greater preference for 14 ppt salinity than the controls. Their finding that thyroid hormone, growth hormone and cortisol all stimulate salinity preference of young Coho salmon, suggested the importance of these hormones in osmoregulation and preparing smolts for migration.^22^

In conclusion according to our results, the thyroid hormones changes in salinity stress in grass carp showed that these hormones may be involved in maintenance of hemostasis in this fish species after salinity changes. Tri-iodothyronine reduction if remain low, may affect body growth or even death of the fish in prolonged exposure to high salinities. Therefore, in this situation parenteral and/or oral administration of T_3 _may increase salinity tolerance. However, for this suggestion it needs more investigation in different ages and conditions.
